# Development and validation of a machine learning model for predicting 30‐day major morbidity and mortality following radical cystectomy: An American College of Surgeons National Surgical Quality Improvement Program study

**DOI:** 10.1002/bco2.70224

**Published:** 2026-05-18

**Authors:** Gurpremjit Singh, Archan Khandekar, Ahmad Abdelaziz, Hemendra N. Shah, Sanoj Punnen, Mark L. Gonzalgo, Dipen J. Parekh

**Affiliations:** ^1^ Desai Sethi Urology Institute University of Miami Miller School of Medicine Miami Florida USA

**Keywords:** artificial intelligence, bladder cancer, cystectomy, machine learning, morbidity, radical cystectomy

## Abstract

**Objective:**

To develop and validate machine learning models for predicting 30‐day major morbidity and mortality in patients undergoing radical cystectomy (RC) using the American College of Surgeons National Surgical Quality Improvement Project (ACS‐NSQIP) and to compare performance against a logistic regression model.

**Material and methods:**

We identified 11 241 patients from the ACS‐NSQIP database 2020–2024 who underwent radical cystectomy. Demographics and comorbidities were extracted along with targeted variables from the NSQIP RC‐targeted database. The cohort was split into a training and an independent validation dataset. Predictive models were developed for logistic regression, random forest (RF) and XGBoost. Model performance was evaluated using the area under the receiver operating characteristic (ROC) curve, SHapley Additive exPlanations (SHAP) analysis, sensitivity, specificity and Brier scores.

**Results:**

Of 11 241 patients, 2691 (23.9%) experienced at least one major complication, and 185 (1.6%) died within 30 days. Overall complications were 6365 (56.62%). Non‐survivors were significantly older (72.65 ± 10.14 vs. 68.50 ± 10.32 years, *p* < 0.001). Patients with major morbidity had lower mean albumin levels (3.92 ± 0.56 vs. 4.01 ± 0.48, *p* < 0.001). Logistic regression identified high BMI (OR 1.15, *p* < 0.001), black race (OR 1.34, *p* = 0.003), Hispanic ethnicity (OR 1.37, *p* = 0.009), prior pelvic surgery (OR 1.15, *p* = 0.002) and continent diversions (OR 1.46, *p* = 0.001) as predictors of 30‐day major morbidity, while low frailty (mFI‐5 ≤ 1; OR 0.72, *p* = 0.001) and higher preoperative albumin (OR 0.88, *p* < 0.001) were protective. For 30‐day mortality, increasing age (OR 1.42, *p* < 0.001) was the strongest risk factor. For 30‐day morbidity, the XGBoost model achieved the highest AUC 0.796 (95% CI: 0.783–0.814). For 30‐day mortality, the RF model showed superior discrimination with an AUC of 0.921 (95% CI: 0.908–0.934). SHAP analysis showed predictors of major morbidity were frailty, BMI and advanced age, whereas predictors of mortality were age, ASA class and preoperative creatinine levels. Decision curve analysis showed net clinical benefit for all three models. The web‐based tool can be accessed and used for prediction (https://cystectomyai.streamlit.app/).

**Conclusions:**

We developed and validated machine learning models for 30‐day major morbidity and 30‐day mortality following radical cystectomy. These findings support the integration of machine learning into clinical workflows to enhance preoperative counselling and personalized risk reduction.

## 1 INTRODUCTION

Radical cystectomy (RC) remains the standard of care for non‐metastatic muscle‐invasive bladder cancer (MIBC) and selected high‐risk non‐muscle‐invasive bladder cancer.^1^ NSQIP analysis from 2006 to 2011 reported overall 30‐day complications around 56% and mortality around 3.2% following cystectomy.^2^ This is among the most morbid operations in urological oncology, with even recent studies showing 30‐day complications of 30%–50% and perioperative mortality of 2%–3%.^3^, ^4^ There is consistently a higher rate of gastrointestinal, infectious and cardiopulmonary events after RC, underscoring the need for accurate perioperative risk stratification.^5^

Existing models for post‐RC morbidity and mortality are based on the American College of Surgeons National Surgical Quality Improvement Program (ACS‐NSQIP) and were developed using a heterogeneous study population, and they do not account for RC‐specific patterns of morbidity and mortality.^5^ Multiple external validation studies show that the ACS‐NSQIP calculator performs poorly in patients undergoing RC.^6^, ^7^ Golan et al., in a study of 945 patients, reported that the ACS‐NSQIP risk calculator had an AUC of 0.69 and concluded that it performed poorly in predicting outcomes after RC.^6^ Lone et al. in a study of 462 patients also showed that the ACS NSQIP risk calculator had AUC values of 0.50–0.64 for most complications and concluded that it performed poorly, highlighting the need for a procedure‐specific risk calculator.^7^

The morbidity and mortality in RC are driven by a complex interaction of baseline burden, nutritional status and perioperative factors. Older age, higher ASA, poor functional status and hypoalbuminaemia have consistently been associated with increased risk of major complications and early death after RC.^8^, ^9^ Recent studies show that enhanced recovery after surgery (ERAS) components help improve short‐term outcomes, but these are not fully utilized in prediction tools.^10^

Machine learning (ML) models offer a solution by utilizing complex non‐linear interactions between variables to improve prediction outcomes. In this study, we utilized the cystectomy‐specific NSQIP database from 2020 to 2024 to develop and validate an ML prediction model for 30‐day morbidity and mortality. We compared the performance of logistic regression, used to develop the ACS‐NSQIP surgical risk calculator, with that of the random forest ML and XGBoost ML models. Our aim was to create an RC‐specific decision‐support tool that provides clinically useful insights into drivers of perioperative risk.

## 2 METHODS

### 2.1 Patient population

**FIGURE 1:**
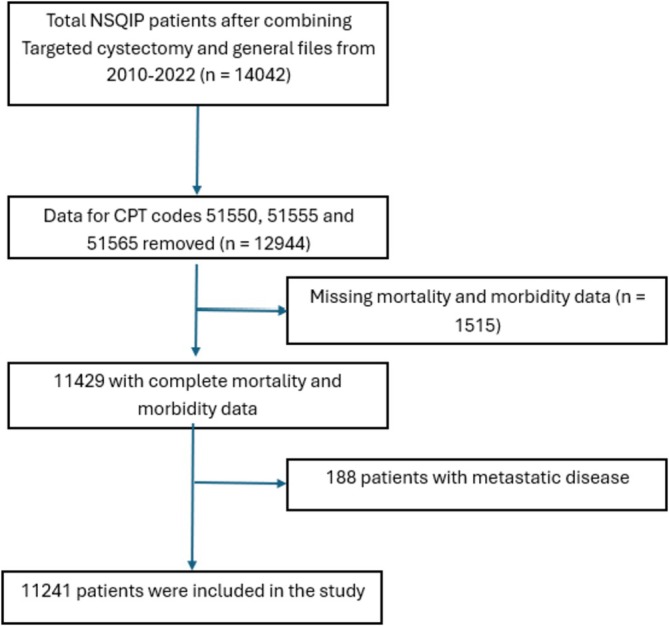
Flow diagram for the sAmerican College of Surgeons National Surgical Quality Improvement Program (ACS‐NSQIP) cohort.

We utilized the ACS‐NSQIP cystectomy‐specific 2020–2024 database for this study. The ACS‐NSQIP Urology procedure‐targeted database, along with the participant user database, was utilized. The targeted database contains more granular variables and outcomes.^11^ We included all patients who underwent cystectomy. We excluded patients who underwent surgery with a purely laparoscopic approach, with metastatic disease, and those who had missing morbidity and mortality data. Figure 1 gives the patient selection flow diagram.

### 2.2 Feature selection for models

We used preoperative and operative variables from the NSQIP database to create predictive models. We used demographic data on age, sex, body mass index (BMI) (calculated from weight and height), race and ethnicity. Clinical features included were American Society of Anesthesiologists (ASA) Physical Classification, smoking history, prior pelvic surgery, prior pelvic radiation, neoadjuvant chemotherapy, acute renal failure, preoperative dialysis, ascites and >10% weight loss. Overall patient frailty was assessed using the five‐factor modified frailty index (mFI‐5), categorized as low (≤1) versus high (≥2).^12^ To prevent multicollinearity, the individual components used to create the mFI‐5 (diabetes mellitus, hypertension requiring medication, history of COPD, congestive heart failure and dependent functional status) were excluded as independent variables for the model. Laboratory values included preoperative albumin, creatinine, haematocrit and platelet count. Surgical factors included surgical approach (robotic vs. open). CPT codes were categorized into simple cystectomy (reference category), RC with ileal conduit, RC with continent diversions and pelvic exenteration.

### 2.3 Outcomes and definitions

The primary outcome was the prediction of 30‐day major morbidity and 30‐day mortality using ML and traditional statistical models. Major morbidity was defined as any Clavien–Dindo ≥3 complications within 30 days using the ACS‐NSQIP general and cystectomy‐specific variables. It included myocardial infarction, cardiac arrest, prolonged ventilation, pneumonia, pulmonary embolism, sepsis, septic shock, rectal injury, ureteral fistula, 30‐day ureteral obstruction, lymphocele requiring intervention/surgery and acute kidney injury leading to dialysis. Cases with missing morbidity and mortality data were excluded.

### 2.4 Statistical analysis

Descriptive statistics were used to define the overall study cohort. Chi‐square was used for categorical variables across groups, and Student's *t*‐test was used for continuous variables. Multivariate logistic regression was used as a baseline to compare ML models. This model served as an NSQIP‐style benchmark.

A stratified 80:20 split was carried out. The training set was used for algorithm training and performance assessment with fivefold cross‐validation. We addressed class imbalance using SMOTE (Synthetic Minority Over‐Sampling Technique) on the training dataset.^13^ Missing data was imputed using k‐nearest neighbours.

We then trained two ensemble ML models for each outcome: a random forest classifier and an extreme gradient boosting (XGBoost) classifier.^14^ Model performance was assessed with discrimination (receiver operating characteristic curve), sensitivity, specificity, Brier score, calibration plots and decision curve analysis (DCA). Model hyperparameters were tuned. Shapley Additive exPlanations (SHAP) were employed to determine feature importance. All analysis was done with Python 3.11, and a web‐based deployment of the best model was done using GitHub and Streamlit.

The study used the TRIPOD‐AI (Transparent Reporting of a Multivariate Prediction Model for Individual Program or Diagnosis—Artificial Intelligence) guidelines for model development and reporting^15^ (Supporting Information).

## 3 RESULTS

Among 11 241 patients who underwent cystectomy, 2691 (23.9%) experienced at least one 30‐day major complication, and 185 (1.6%) died within 30 days. Patients with major morbidity had a similar mean age to those without (68.47 ± 10.84 vs. 68.60 ± 10.16 years, *p* = 0.563) but were more comorbid. Non‐survivors were significantly older (72.65 ± 10.14 vs. 68.50 ± 10.32 years, *p* < 0.001) and had a higher prevalence of ASA class 4 (17.3% vs. 6.9%, *p* < 0.001). Preoperative laboratory values showed that patients with major morbidity had lower albumin (3.92 ± 0.56 vs. 4.01 ± 0.48, *p* < 0.001) and haematocrit (36.67 ± 5.97 vs. 37.01 ± 5.91, *p* = 0.010). Table 1 summarizes demographic and baseline characteristics.

**TABLE 1:** Demographics and baseline characteristics.

	Total (*N* = 11 241)	Major morbidity (*n* = 2691)	No major morbidity (*n* = 8550)	*p*‐values (morbidity)	30‐day mortality (185)	Survived (11056)	*p*‐value (mortality)
Demographics							
Age (years), mean ± SD	68.57 ± 10.33	68.47 ± 10.836	68.60 ± 10.163	0.563	72.65 ± 10.14	68.50 ± 10.32	**<0.001**
BMI (kg/m^2^), mean ± SD	28.43 ± 5.72	29.15 ± 6.22	28.21 ± 5.53	**<0.001**	27.70 ± 6.28	28.45 ± 5.71	0.081
Sex, *n* (%)				0.479			0.356
Male	8485 (75.5%)	2045 (76.0%)	6440 (75.3%)		145 (78.4%)	8340 (75.4%)	
Female	2756 (24.5%)	646 (24.0%)	2110 (24.7%)		40 (21.6%)	2716 (24.6%)	
Hispanic *n* (%)	373 (4.0%)	112 (5.0%)	261 (3.7%)	**0.003**	4 (2.6%)	369 (4.0%)	0.375
Race, *n* (%)				**0.001**			0.190
White	8366 (90.5%)	1946 (88.9%)	6420 (91.0%)		129 (86.0%)	8237 (90.6%)	
Black	566 (6.1%)	168 (7.7%)	398 (5.6%)		12 (8.0%)	554 (6.1%)	
Asian	196 (2.1%)	41 (1.9%)	155 (2.2%)		5 (3.3%)	191 (2.1%)	
Mixed	115 (1.2%)	35 (1.6%)	80 (1.1%)		4 (2.7%)	111 (1.2%)	
Current smoker, *n* (%)	2238 (19.9%)	530 (19.7%)	1708 (20.0%)	0.750	35 (18.9%)	2203 (19.9%)	0.734
Frailty index				**<0.001**			**<0.001**
Low (≤1)	8640 (76.9%)	1901 (70.6%)	6739 (78.8%)		114 (61.6%)	8526 (77.1%)	
High (≥2)	2601 (23.1%)	790 (29.4%)	1811 (21.2%)		71 (38.4%)	2530 (22.9%)	
Comorbidities, *n* (%)							
Diabetes mellitus				**<0.001**			**0.004**
Insulin dependent	768 (6.8%)	253 (9.4%)	515 (6.0%)		24 (13.0%)	744 (6.7%)	
Non‐insulin dependent	1566 (13.9%)	416 (15.5%)	1150 (13.5%)		25 (13.5%)	1541 (13.9%)	
Functional status				**<0.001**			**<0.001**
Partial dependence	258 (2.3%)	105 (3.9%)	153 (1.8%)		14 (7.6%)	244 (2.2%)	
Total dependence	47 (0.4%)	20 (0.7%)	27 (0.3%)		1 (0.5%)	46 (0.4%)	
History of COPD	756 (6.7%)	228 (8.5%)	528 (6.2%)	**<0.001**	19 (10.3%)	737 (6.7%)	0.052
Congestive heart failure	396 (3.5%)	134 (5.0%)	262 (3.1%)	**<0.001**	17 (9.2%)	379 (3.4%)	**<0.001**
Hypertension meds	6541 (58.2%)	1700 (63.2%)	4841 (56.6%)	**<0.001**	133 (71.9%)	6408 (58.0%)	**<0.001**
Chronic steroid use	605 (5.4%)	172 (6.4%)	433 (5.1%)	**0.008**	14 (7.6%)	591 (5.3%)	0.184
Weight loss (>10% in 6 months)	114 (1.0%)	26 (1.0%)	88 (1.0%)	0.776	5 (2.7%)	109 (1.0%)	**0.021**
Preoperative laboratory values							
Albumin (mg/dL), mean ± SD	3.99 ± 0.51	3.92 ± 0.56	4.01 ± 0.48	**<0.001**	3.77 ± 0.60	3.99 ± 0.50	**<0.001**
WBC (10^3^/μL), mean ± SD	7.84 ± 3.55	8.02 ± 3.71	7.78 ± 3.49	**0.003**	8.50 ± 4.81	7.82 ± 3.52	**0.006**
Haematocrit (%), mean ± SD	36.93 ± 5.92	36.67 ± 5.97	37.01 ± 5.91	**0.01**	35.50 ± 6.16	36.95 ± 5.92	**<0.001**
Platelets (×10^9^/L), mean ± SD	254.81 ± 99.32	257.35 ± 102.67	254.02 ± 98.23	0.136	256.84 ± 112.91	254.78 ± 99.08	0.391
Total bilirubin (mg/dL)	0.48 ± 0.32	0.47 ± 0.27	0.48 ± 0.33	0.161	0.52 ± 0.31	0.48 ± 0.32	**0.043**
Creatinine (mg/dL), mean ± SD	1.21 ± 0.72	1.21 ± 0.67	1.21 ± 0.73	0.848	1.43 ± 1.03	1.21 ± 0.71	**<0.001**
BUN (mg/dL), mean ± SD	21.39 ± 10.34	21.35 ± 10.84	21.40 ± 10.18	0.814	24.78 ± 14.28	21.33 ± 10.25	**<0.001**
INR, mean ± SD	1.06 ± 0.22	1.08 ± 0.29	1.06 ± 0.19	**<0.001**	1.07 ± 0.14	1.06 ± 0.22	0.323
Preoperative							
Preoperative transfusion, *n* (%)	190 (1.7%)	62 (2.3%)	128 (1.5%)	**0.005**	8 (4.3%)	182 (1.6%)	**0.005**
Neoadjuvant chemotherapy, *n* (%)	4012 (35.7%)	936 (34.8%)	3076 (36.0%)	0.26	56 (30.3%)	3956 (35.8%)	0.121
ASA classification, *n* (%)				**<0.001**			**<0.001**
ASA 1	17 (0.2%)	2 (0.1%)	15 (0.2%)		0 (0.0%)	17 (0.2%)	
ASA 2	1953 (17.4%)	386 (14.3%)	1567 (18.3%)		8 (4.3%)	1945 (17.6%)	
ASA 3	8462 (75.3%)	2078 (77.2%)	6384 (74.7%)		143 (77.3%)	8319 (75.2%)	
ASA 4	799 (7.1%)	221 (8.2%)	578 (6.8%)		32 (17.3%)	767 (6.9%)	
ASA 5	3 (<0.1%)	3 (0.1%)	0 (0.0%)		2 (1.1%)	1 (<0.1%)	
Mechanical bowel preparation, *n* (%)	2274 (20.2%)	580 (21.6%)	1694 (19.8%)	**0.05**	37 (20.0%)	2237 (20.2%)	0.938
Oral antibiotic preparation, *n* (%)	869 (7.7%)	245 (9.1%)	624 (7.3%)	**0.002**	12 (6.5%)	857 (7.8%)	0.523
Prior pelvic surgery, *n* (%)	5743 (51.1%)	1440 (53.5%)	4303 (50.3%)	**0.004**	90 (48.6%)	5653 (51.1%)	0.503

*Note*: The *p*‐values calculated using Pearson chi‐square for categorical variables and independent samples *t*‐test for continuous variables.

Abbreviations: ASA, American Society of Anesthesiologists; BMI, body mass index; DM, diabetes mellitus; INR, international normalized ratio; WBC, white blood cell.

### 3.1 Operative details and postoperative outcomes

Mean operative time for the entire cohort was 340.25 ± 118.48 min. Patients who experienced major morbidity had longer operative times (361.88 ± 130.57 vs. 333.44 ± 113.57 min, *p* < 0.001). Open surgery was performed in 7383 (65.7%) patients, and a robotic approach was used in 3858 (34.3%) of the patients. The total length of hospital stay was also prolonged in major morbidity (10.45 ± 6.98 vs. 6.95 ± 4.02 days, *p* < 0.001) and in the mortality cohort (10.94 ± 8.64 vs. 7.69 ± 4.96 days, *p* < 0.001). Table 2 summarizes the perioperative and operative findings.

**TABLE 2:** Operative, postoperative outcomes, complications and cause specific events.

Outcome, *n* (%)	Total (*n* = 11 241)	30‐day major morbidity (*n* = 2691)	No major morbidity (*n* = 8550)	*p*‐values(morbidity)	30‐day mortality (*n* = 185)	Survived (*n* = 11 056)	*p*‐value (mortality)
Surgery type				**0.020**			0.061
Open	7383 (65.7%)	1818 (67.6%)	5565 (65.1%)		134 (72.4%)	7249 (65.6%)	
Robotic	3858 (34.3%)	873 (32.4%)	2985 (34.9%)		51 (27.6%)	3807 (34.4%)	
Operative							
Operative time (min), mean ± SD	340.25 ± 118.48	361.88 ± 130.57	333.44 ± 113.57	**<0.001**	332.09 ± 119.24	340.38 ± 118.46	0.345
Surgical drain placement, *n* (%)	10 765 (95.8%)	2588 (96.2%)	8177 (95.6%)	0.229	176 (95.1%)	10 589 (95.8%)	0.668
Total length of stay (days), *Mean ± SD*	7.74 ± 5.06	10.45 ± 6.98	6.95 ± 4.02	**<0.001**	10.94 ± 8.64	7.69 ± 4.96	**<0.001**
Discharge opioids, *n* (%)				**<0.001**			**<0.001**
Monotherapy	1556 (13.8%)	306 (11.4%)	1250 (14.6%)		6 (3.2%)	1550 (14.0%)	
Dual therapy	20 (0.2%)	5 (0.2%)	15 (0.2%)		0 (0.0%)	20 (0.2%)	
Poly opioid	63 (0.6%)	25 (0.9%)	38 (0.4%)		0 (0.0%)	63 (0.6%)	
Total discharge MME, mean ± SD	N/A	9.23 ± 48.73	9.73 ± 40.91	0.596	3.57 ± 27.88	9.71 ± 43.11	0.053
Wound complications, *n* (%)							
Superficial SSI	469 (4.2%)	134 (5.0%)	335 (3.9%)	**0.016**	5 (2.7%)	464 (4.2%)	0.313
Deep incisional SSI	80 (0.7%)	51 (1.9%)	29 (0.3%)	**<0.001**	1 (0.5%)	79 (0.7%)	0.78
Organ/space SSI	966 (8.6%)	966 (35.9%)	0 (0.0%)	**<0.001**	39 (21.1%)	927 (8.4%)	**<0.001**
Wound dehiscence	201 (1.8%)	201 (7.5%)	0 (0.0%)	**<0.001**	8 (4.3%)	193 (1.7%)	**0.009**
Cystectomy specific complications, *n* (%)							
Ureteral obstruction (30 days)	559 (5.0%)	420 (15.6%)	139 (1.6%)	**<0.001**	10 (5.4%)	549 (5.0%)	0.43
Ureteral fistula	441 (3.9%)	441 (16.4%)	0 (0.0%)	**<0.001**	12 (6.5%)	429 (3.9%)	0.07
Lymphocele/collections				**<0.001**			**<0.001**
Conservative management	360 (3.2%)	173 (6.4%)	187 (2.2%)		7 (3.8%)	353 (3.2%)	
Intervention required	251 (2.2%)	251 (9.3%)	0 (0.0%)		3 (1.6%)	248 (2.2%)	
Reoperation required	32 (0.3%)	32 (1.2%)	0 (0.0%)		8 (4.1%)	28 (0.2%)	
Gastrointestinal events, *n* (%)							
*Clostridioides difficile* infection	270 (2.4%)	138 (5.1%)	132 (1.5%)	**<0.001**	8 (4.3%)	262 (2.4%)	0.085
Anastomotic bowel leak requiring intervention	311 (2.8%)	311 (11.6%)	0 (0.0%)	**<0.001**	23 (12.4%)	288 (2.6%)	**<0.001**
NG tube placement	2028 (18.0%)	961 (35.7%)	1067 (12.5%)	**<0.001**	81 (43.8%)	1947 (17.6%)	**<0.001**
Renal complications, *n* (%)				**<0.001**			**<0.001**
Acute kidney injury (AKI)	49 (0.4%)	43 (1.6%)	6 (0.1%)		16 (8.6%)	33 (0.3%)	
Postop dialysis	60 (0.5%)	60 (0.5%)	0		24 (13.0%)	36 (0.3%)	
Systemic complications, *n* (%)							
Sepsis	734 (6.5%)	734 (27.3%)	0 (0.0%)	NA	725 (6.6%)	9 (4.9%)	**0.355**
Septic shock	316 (2.8%)	316 (11.7%)	0 (0.0%)	NA	239 (2.2%)	77 (41.6%)	**<0.001**
DVT	287 (2.6%)	287 (10.7%)	0 (0.0%)	NA	277 (2.5%)	10 (5.4%)	**0.013**
Pneumonia	310 (2.8%)	310 (11.5%)	0 (0.0%)	NA	260 (2.4%)	50 (27%)	**<0.001**
Pulmonary embolism	159 (1.4%)	159 (5.9%)	0 (0.0%)	NA	149 (1.3%)	10 (5.4%)	**<0.001**
Myocardial infarction	156 (1.4%)	156 (5.8%)	0 (0.0%)	NA	129 (1.2%)	<27 (14.6%)	**<0.001**
Stroke/CVA	42 (0.4%)	42 (1.6%)	0 (0.0%)	NA	38 (0.3%	38 (0.3%)	**<0.001**

*Note*: The *p*‐values calculated using Pearson chi‐square for categorical variables and independent samples *t*‐test for continuous variables. Values in bold indicate statistical significance at the *p* < 0.05 level.

Abbreviations: DVT, deep vein thrombosis; mFI‐5, modified five‐item frailty index (includes diabetes, functional status, COPD, CHF and hypertension); MME, morphine milligram equivalents; NG, nasogastric; PE, pulmonary embolism; SSI, surgical site infection; NA, Not applicable.

Overall complications of the whole cohort were 6365 (56.62%). Among postoperative complications, organ/space surgical site infections were 8.6%, sepsis was present in 6.5%, cystectomy‐related events like lymphocele were present in 5.7%, and anastomotic bowel leak was present in 2.8% of patients. Table 2 lists all the complications.

### 3.2 Multivariate logistic regression

Multivariate logistic regression analysis (Table 3) was performed to identify the independent predictors of 30‐day outcomes. For major morbidity, high BMI (OR 1.15, *p* < 0.001), Black race (OR 1.34, *p* = 0.003), Hispanic ethnicity (OR 1.37, *p* = 0.009), prior pelvic surgery (OR 1.15, *p* = 0.002) and continent diversions (OR 1.46, *p* = 0.001) were risk drivers, while low frailty (mFI‐5 ≤ 1; OR 0.72, *p* = 0.001) and higher preoperative albumin (OR 0.88, *p* < 0.001) were protective. For 30‐day mortality, increasing age (OR 1.42, *p* < 0.001) was the strongest risk factor. Low frailty (OR 0.30, *p* < 0.001), robotic surgery (OR 0.616, *p* = 0.007) and higher preoperative albumin (OR 0.79, *p* = 0.007) were protective factors.

**TABLE 3:** Multivariate logistic regression analysis of 30‐day major morbidity and mortality.

Variable (reference group)	Major morbidity OR (95% CI)	*p*‐value	30‐day mortality OR (95% CI)	*p*‐value
Demographics				
Age (per year increase)	1.01 (0.97–1.06)	0.564	1.42 (1.17–1.71)	**<0.001**
BMI (kg/m^2^)	1.15 (1.09–1.21)	**<0.001**	0.89 (0.75–1.04)	0.093
Sex: Female (vs. male)	0.94 (0.84–1.05)	0.253	0.96 (0.66–1.41)	0.845
Race (vs. White)				
Black	1.34 (1.11–1.63)	**0.003**	0.99 (0.51–1.90)	0.972
Asian	0.94 (0.66–1.34)	0.735	1.05 (0.35–3.13)	0.936
AIAN/Native/Other	1.14 (0.74–1.73)	0.557	1.07 (0.27–4.27)	0.921
Ethnicity				
Hispanic (vs. non‐Hispanic)	1.37 (1.08–1.74)	**0.009**	0.83 (0.34–2.07)	0.695
Frailty status				
mFI‐5 low (vs. high ≥2)	0.72 (0.58–0.88)	**0.001**	0.30 (0.16–0.54)	**<0.001**
Clinical context				
Neoadjuvant chemotherapy	0.95 (0.86–1.05)	0.348	0.89 (0.63–1.25)	0.498
Prior pelvic surgery	1.15 (1.05–1.26)	**0.002**	0.95 (0.70–1.28)	0.739
Prior pelvic radiation	1.13 (0.98–1.29)	0.085	0.94 (0.61–1.46)	0.796
Current smoker	1.03 (0.92–1.15)	0.643	0.97 (0.65–1.44)	0.871
Laboratory values				
Preoperative albumin (g/dL)	0.88 (0.84–0.92)	**<0.001**	0.79 (0.69–0.91)	**0.007**
Preoperative creatinine	0.96 (0.91–1.02)	0.214	1.08 (0.94–1.25)	0.281
Preoperative haematocrit (%)	0.97 (0.93–1.03)	0.334	0.95 (0.80–1.12)	0.518
Preoperative platelets	1.02 (0.97–1.07)	0.446	1.03 (0.90–1.19)	0.661
Surgical factors				
Robotic surgery (vs. open)	0.932 (0.844–1.028)	0.16	0.616 (0.434–0.875)	**0.007**
Radical cystectomy with ileal conduit	1.010 (0.814–1.254)	0.928	0.709 (0.379–1.328)	0.283
Radical cystectomy with continent diversions	1.466 (1.167–1.842)	**0.001**	0.478 (0.206–1.107)	0.085
Pelvic exenteration	0.948 (0.725–1.239)	0.695	0.713 (0.313–1.624)	0.420

*Note:* Values in bold indicate statistical significance at the *p* < 0.05 level.

### 3.3 ML models

On the independent validation set, ML models demonstrated superior discrimination compared to logistic regression.

For 30‐day mortality, the random forest algorithm achieved the highest performance with an AUC of 0.921 (95% CI: 0.908–0.934). The random forest yielded a sensitivity of 90.1% and a specificity of 89.8% with excellent overall model fit (Brier score: 0.145).

For 30‐day morbidity, the XGBoost algorithm achieved the highest accuracy, showing a validation set AUC of 0.796 (95% CI: 0.783–0.814). The model showed a sensitivity of 55.1% but a high specificity of 90.8% with a Brier score of 0.177. Table 4 summarizes all the model parameters.

**TABLE 4:** Predictive performance for various models.

Model	Training set AUC (80%; *n* = 8993)	Fivefold cross‐validation set	Validation set AUC (20%; *n* = 2248)	Sensitivity	Specificity	Brier score
Predictor—30‐day mortality						
Logistic regression	0.752	0.749	0.751 (0.732–0.770)	0.799	0.579	0.199
Random forest	0.929	0.925	0.921 (0.908–0.934)	0.901	0.898	0.145
XGBoost	0.808	0.803	0.802 (0.781–0.823)	0.861	0.883	0.188
Predictor—30‐day morbidity						
Logistic regression	0.601	0.592	0.594 (0.570–0.620)	0.525	0.601	0.236
Random forest	0.809	0.751	0.760 (0.740–0.780)	0.639	0.725	0.207
XGBoost	0.828	0.789	0.796 (0.783–0.814)	0.551	0.908	0.177

### 3.4 Clinical interpretability and web‐based calculator

**FIGURE 2:**
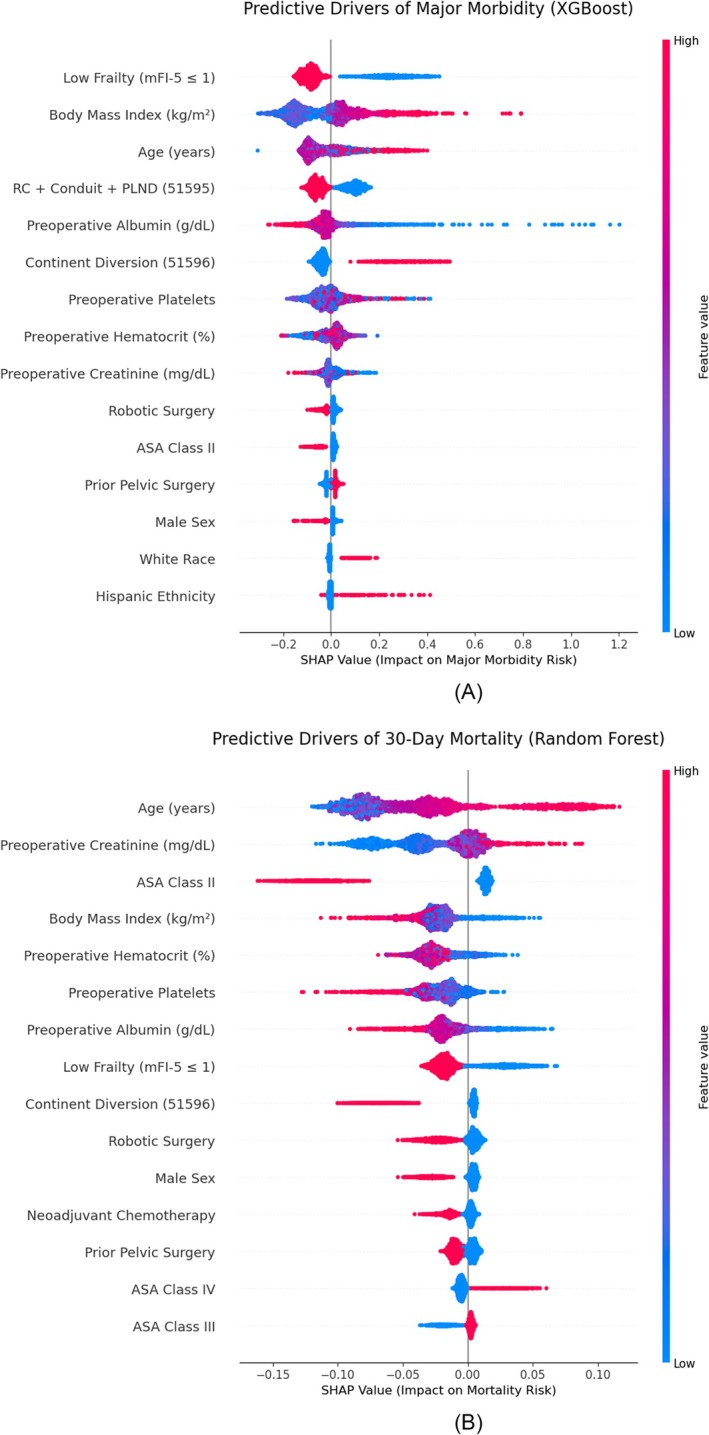
(A) SHAP (Shapley additive exPlanations) summary plot for 30‐day morbidity following radical cystectomy. (B) SHAP (Shapley additive exPlanations) summary plot for 30‐day mortality following radical cystectomy.

SHAP analysis was used to identify the primary drivers of 30‐day morbidity (Figure 2A) and 30‐day mortality (Figure 2B). For 30‐day major morbidity, the primary drivers were the frailty index, BMI and age. High BMI and complexity of continent diversions increase the SHAP score's predictive value for morbidity. The random forest revealed that the 30‐day mortality was primarily driven by age, preoperative creatinine levels, ASA class and BMI. Robotic surgery and low frailty were associated with negative SHAP scores, showing a protective effect. The web‐based tool can be accessed and used for prediction. (https://cystectomyai.streamlit.app/).

### 3.5 Calibration and DCA

**FIGURE 3:**
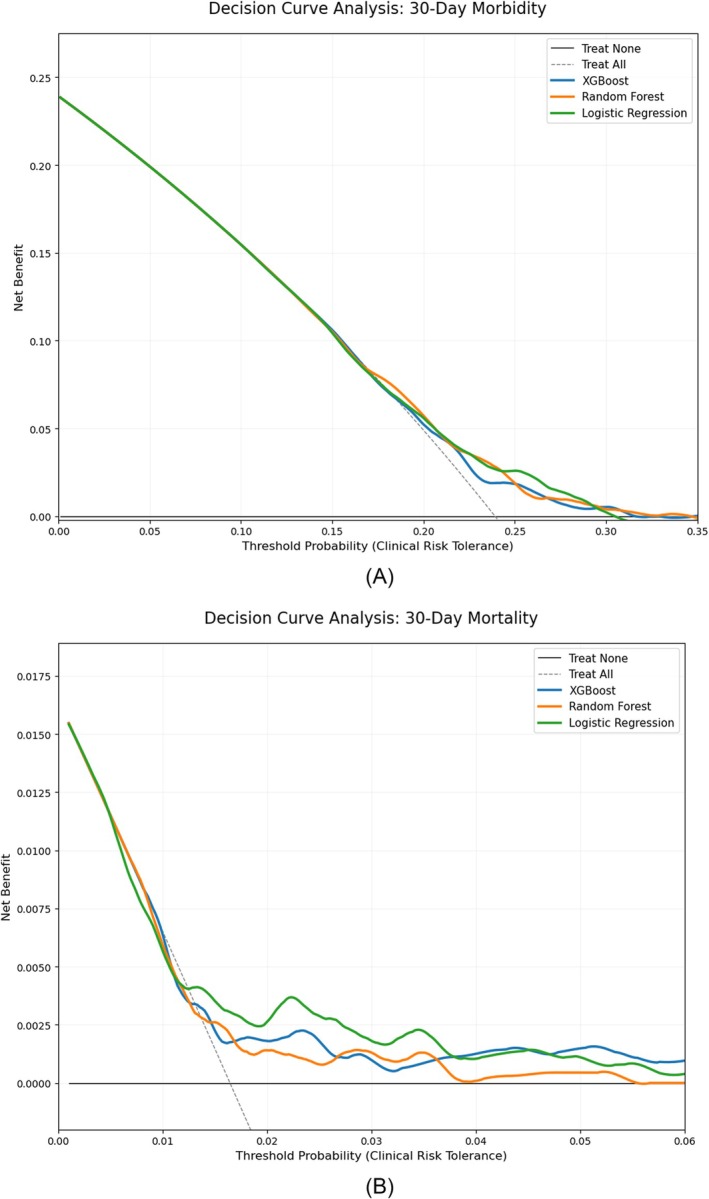
Decision curve analysis (DCA) evaluating the net clinical benefit. (A) Morbidity. (B) Mortality.

For 30‐day morbidity, the ML models showed better calibration than the logistic regression model. For 30‐day mortality, the XGBoost showed better calibration than other models (Figures **S1A** and **S1B**). All three models showed net clinical benefit for 30‐day mortality and 30‐day morbidity on DCA in a 1%–6% range for mortality and a 15%–30% range for major morbidity (Figure 3A,B).

## 4 DISCUSSION

We developed and validated ML models to predict 30‐day morbidity and mortality, outperforming traditional logistic regression models used in ACS‐NSQIP surgical risk calculations. The random forest model achieved excellent discrimination (AUC: 0.93) with good overall performance (Brier score: 0.15), while the XGBoost model showed strong discrimination (AUC: 0.80) and good performance (Brier score: 0.18) for 30‐day mortality. All the models showed a net clinical benefit. These findings support the development of ML models specific to RC.

Despite being a highly comorbid procedure, no robust predictive model has been reported in the literature. Several studies have shown limitations of ACS‐NSQIP prediction in the cystectomy population. Golan et al. and others reported fair AUC and poor calibration of the NSQIP risk calculator.^6^, ^9^ Our results with logistic regression models also show similar findings. Taylor et al. developed an ML model to predict postoperative complications after RC. However, their model also performed poorly with an AUC of 0.63. Their model used the 2005–2016 NSQIP database and lacked cystectomy‐specific variables as given in the targeted cystectomy database of NSQIP.^16^ We used a cystectomy‐specific database to develop a more robust model, which we incorporated into a public web calculator.

Combined analysis from logistic regression and ML showed that the major predictors of morbidity were frailty (mFI‐5), BMI, age, continent diversions and preoperative albumin. Minor predictors were Black race, Hispanic ethnicity and a history of prior pelvic surgery. For 30‐day mortality, the combined analysis showed that age, BMI, ASA classification, baseline frailty and preoperative creatinine were major predictors. Both methodologies showed that robotic surgery and higher preoperative albumin were protective. These findings, particularly regarding the roles of age, frailty and preoperative albumin, show the importance of preoperative optimization and comprehensive geriatric assessment. Chappidi et al. also showed that the modified frailty index can identify patients at greatest risk for severe morbidity and mortality.^17^ We used the mFI‐5 score, which has been developed as a geriatric evaluation tool with reduced variables to simplify data collection.^12^ Preoperative hypoalbuminaemia is associated with increased morbidity and mortality post RC.^18^ Patients with metabolic syndrome are also more likely to have worse perioperative outcomes following RC.^19^

Our XGBoost and logistic regression showed that continent diversions carry significantly higher morbidity than ileal conduits. Joice et al. showed that continent diversion was associated with more readmissions and increased hospital costs. Such patients were more likely to have infectious and genitourinary complications.^20^ Continent reconstructions require longer operative times, more extensive bowel handling and complex anastomoses, which increase the risk of major morbidity.^21^

Our analysis also showed that robotic surgery is an independent protective factor against 30‐day mortality. The RAZOR trial demonstrated non‐inferiority of the open and robotic approaches. They showed similar adverse events (67% in robotic vs. 69% in open), cancer‐specific mortality (19% in robotic vs. 21% in open) and non‐cancer‐specific mortality (7% vs. 7%) in both groups.^22^ The iROC trial showed that 90‐day mortality was 1.2% in the robotic group versus 2.6% in the open surgery group. Although it was lower in the robotic surgery group, the difference was not statistically significant. They showed that days alive and out of hospital within 90 days favoured robotic by 2 days (82 vs. 80, *p* = 0.01).^23^

The overall 30‐day complication rate in this cohort was 56.6%, whereas major complications were 23.9%. The overall complication rate is similar to the NSQIP cohorts from 2006 to 2011, at 56%.^2^ The integration of ERAS protocols has become standard of care for cystectomy, yet their impact on major complications remains unclear. Zhang et al., in a systematic review and meta‐analysis, showed that while ERAS reduced length of stay and time to regular diet, it did not significantly reduce major complications (OR 0.91, *p* = 0.65) or mortality (OR 0.69, *p* = 0.49).^24^ Individual patient data meta‐analysis by Williams et al. showed that ERAS decreased 30‐day and 90‐day overall morbidity without affecting mortality.^25^ Pfail et al. showed in the 2019–2021 NSQIP cohort that adherence with ERAS protocols improved postoperative outcomes. However, only 20% of the cohort received five interventions of ERAS.^10^ The overall morbidity of this surgical procedure, even after two decades, remains almost similar, maybe due to non‐uniform use of ERAS practices.

While traditional regression analysis is the foundation of medical research, it assumes a linear relationship between variables and cannot capture non‐linear interactions. ML algorithms stand out in capturing such interactions.^14^ ML has distinct advantages in tabular datasets. They have superior performance, which can improve preoperative risk assessment. Additionally, it can uncover features and help to personalize risk assessment, which can support shared decision‐making.^14^, ^26^

Several limitations are also present in this study. First, NSQIP is an observational registry limited to 30‐day follow‐up, so we have limited ability to develop long‐term models. Second, unmeasured factors also lead to residual confounding, including ERAS adherence. Third, although these data were cross‐validated and further validated on a separate holdout set, external validation in non‐NSQIP cohorts is needed for broad clinical use. Fourth, many variables, such as socio‐economic status, are missing in NSQIP, which can help in better assessment of various models. Fifth, NSQIP is a database from academic centres, so this model also needs external validation in non‐academic centres prior to clinical use in non‐academic centres. Future scope could include external validation and updating the model based on new metrics. Prospective implementation studies on ERAS and quality improvement can help develop better decision support tools.

## 5 CONCLUSION

We developed and validated two ML models to predict 30‐day major morbidity and mortality post RC. The models showed an AUC of 0.80 and 0.92 for morbidity and mortality, respectively. Additionally, combined regression and SHAP analysis showed that frailty, age, BMI, continent diversion and preoperative nutritional deficiencies were major predictors of morbidity, and age, BMI, ASA classification and frailty were major predictors of mortality.

## AUTHOR CONTRIBUTIONS

*Conception and design*: Gurpremjit Singh. *Data analysis and interpretation*: Gurpremjit Singh and Archan Khandekar. *Critical revision of the manuscript for scientific and factual content*: Gurpremjit Singh, Archan Khandekar, Sanoj Punnen, Mark L. Gonzalgo, Dipen J. Parekh and Hemendra N. Shah. *Drafting the manuscript*: Gurpremjit Singh and Ahmad Abdelaziz. *Supervision*: Archan Khandekar, Hemendra N. Shah and Gurpremjit Singh. *Statistical analysis*: Gurpremjit Singh and Archan Khandekar.

## CONFLICT OF INTEREST STATEMENT

The authors have no conflict of interest to declare.

## ETHICS STATEMENT

The study uses de‐identified NSQIP data and was exempt from institutional review board review.

## Supporting information


**Figure S1A:** Calibration curve assessing the reliability of the prediction model; 1A: Mortality.

**Figure S1B.** Calibration curve assessing the reliability of the prediction model; 1B: Morbidity.

**Data S1.** TRIPOD‐AI checklist.

## Data Availability

Data used for this study are available from ACS NSQIP.
