# Supplementary figures and images for: Development and validation of a machine learning model for predicting 30‐day major morbidity and mortality following radical cystectomy: An American College of Surgeons National Surgical Quality Improvement Program study
Source: BJUI Compass. 2026 May 18;7(5):e70224. doi: 10.1002/bco2.70224 (PMC13183596; doi:10.1002/bco2.70224)

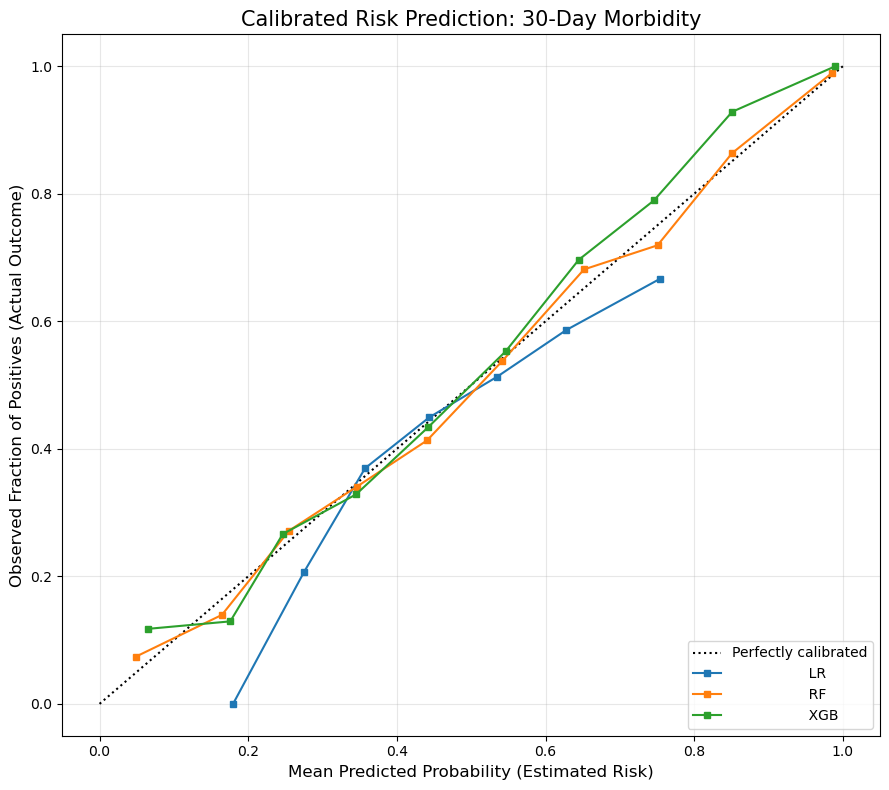

Supplement: Supplementary file 1 — Figure S1A: Calibration curve assessing the reliability of the prediction model; 1A: Mortality. [file BCO2-7-e70224-s002.png]

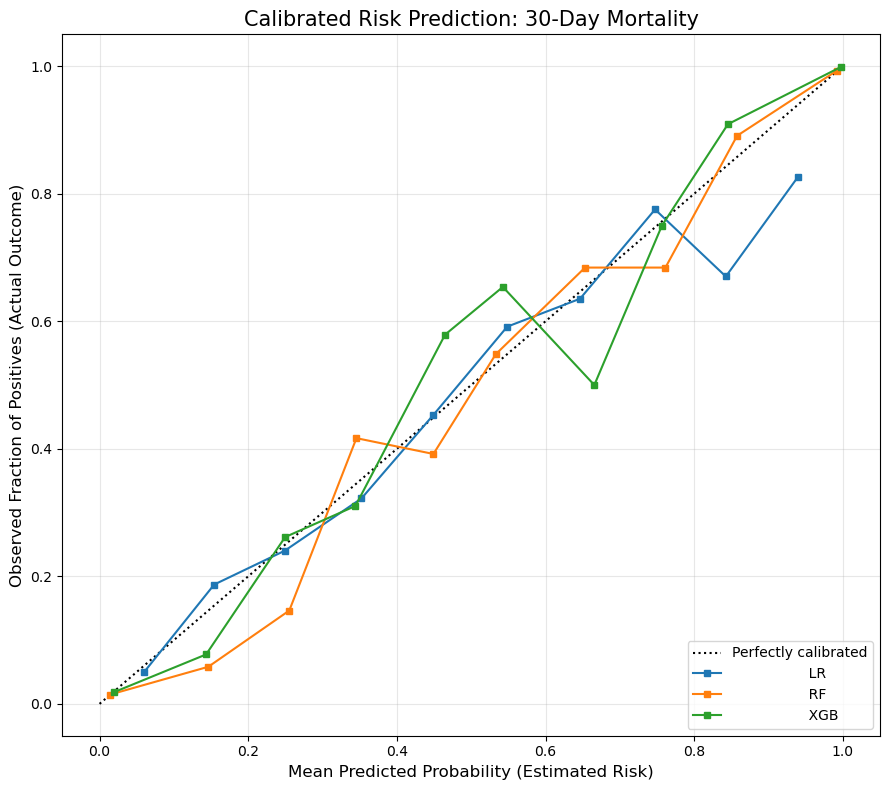

Supplement: Supplementary file 2 — Figure S1B. Calibration curve assessing the reliability of the prediction model; 1B: Morbidity. [file BCO2-7-e70224-s003.png]
